# Mathematical Modeling of Liver Metabolic Activity Under Ex Vivo Conditions upon Exposure to Magnetic Nanoparticles

**DOI:** 10.3390/biomedicines14091877

**Published:** 2026-08-22

**Authors:** Yuliya A. Yakovleva, Konstantin V. Shadrin, Vera G. Pakhomova, Alexander P. Rupenko, Vladimir F. Pyankov, Olga V. Kryukova, Roman N. Yaroslavstev, Marina S. Apanovich, Sergey V. Stolyar

**Affiliations:** 1 Federal Research Center “Krasnoyarsk Science Center” of the Siberian Branch of the Russian Academy of Sciences, Krasnoyarsk 660036, Russia; 2 Department of Medical Cybernetics and Informatics, V.F. Voino-Yasenetsky Krasnoyarsk State Medical University, Krasnoyarsk 660022, Russia

**Keywords:** ex vivo perfusion, liver, flow model, optimal power law, nanoparticles, magnetite

## Abstract

**Background:** In liver transplantation, maintaining donor organ viability is a critical factor determining transplant outcomes. The aim of this study was to evaluate liver metabolic activity under ex vivo perfusion in the presence of magnetite magnetic nanoparticles using mathematical modeling. **Methods:** Ex vivo liver perfusion was performed with nanoparticles added to the perfusate; the concentration of nanoparticles in the inflowing and outflowing perfusate was determined by mass spectrometry. A stoichiometric model of hepatic metabolic fluxes was constructed, and the distribution of energy resources was assessed using the Zipf–Pareto law and its linear approximation, the Zipf–Pareto–Mandelbrot distribution. **Results:** It was shown that nanoparticle uptake by the liver increased from 26.5% to 54.4% over the course of perfusion. The introduction of magnetic nanocomposites altered metabolic fluxes, including glycolysis, the respiratory chain, and oxygen exchange across the surface, leading to redistribution of energy resources within liver cells. Zipf–Pareto analysis showed that energy was optimally distributed among all metabolic fluxes both in the control condition and in the presence of nanoparticles. **Conclusions:** Magnetic nanoparticles alter liver metabolic activity, but the functional state of the organ remains intact.

## 1. Introduction

In liver transplantation, the quality of donor organ preservation is one of the key factors determining both early graft function and long-term outcomes for the recipient [1,2,3]. Although expansion of the donor pool through the use of extended-criteria donor organs has partially alleviated the shortage of donor organs [1], such grafts are more susceptible to ischemic injury, impaired primary graft function, and reduced long-term viability after transplantation [2,3]. In particular, ischemia–reperfusion injury remains one of the central mechanisms underlying early graft dysfunction and primary nonfunction, which may compromise the immediate recovery of organ function and increase the need for urgent retransplantation [1,4,5]. Since early graft injury is also associated with subsequent complications and decreased survival in a proportion of recipients [6], preserving organ viability while minimizing ischemic stress remains one of the major unresolved challenges in modern liver transplantation [7,8].

Static cold storage remains the most widely used method for donor liver preservation [9], as it is technically straightforward to implement [10], logistically convenient, and broadly applied in clinical practice [11,12]. However, this approach is associated with ischemic stress and limits the ability to monitor and modify organ condition during the period between procurement and transplantation [13]. Consequently, machine perfusion, or ex vivo perfusion, has gained increasing attention as an alternative preservation strategy. This technique enables the organ to be maintained under more physiological conditions, allows continuous assessment of viability parameters, and has the potential to extend the duration of safe preservation prior to transplantation [14,15,16]. Nevertheless, there are situations in which cold storage cannot be avoided, for example during long-distance transportation of the organ or when organizational constraints prevent the implementation of perfusion at the procurement site. Insufficiently optimized preservation conditions, as well as the forced combination of different preservation strategies (cold storage and/or machine perfusion) due to logistical and organizational limitations, may lead to a range of complications, including ischemic injury, early graft dysfunction, and delayed graft function [14]. In addition, donor organ damage may occur as a result of ice crystal formation caused by non-uniform freezing and thawing during cryopreservation, which is particularly relevant when working with organs obtained from extended-criteria donors [17,18,19].

In such cases, magnetic nanoparticles, such as iron oxide nanoparticles, are considered for donor organ restoration and preservation. These particles can act as cryoprotective agents and prevent ice crystal formation [20,21], as well as exhibit properties that may enhance graft viability during ex vivo perfusion [22]. These nanoparticles may influence the thermodynamics of the surrounding medium, reduce membrane damage during freezing and thawing [20,21], and potentially improve tissue stability and microcirculation under perfusion conditions [23].

When using nanocomposites in ex vivo perfusion, the question of their impact on the liver arises, since nanomaterials are capable of influencing the metabolism of organ cells [24,25,26]. To exclude the possibility of incorrect functional functioning of the organ, it is necessary to ensure an adequate supply of energy to its cells [21,27]. However, if the energy distribution is unbalanced, this may lead to metabolic disorders [28]. One such disorder caused by the addition of nanoparticles may be a misalignment of the distribution of intracellular energy resources [25,29].

To assess the metabolic activity of cells and the quality of organ functioning under ex vivo conditions, physiological parameters (peripheral vascular resistance [30,31], bile production rate [5,32,33]) are determined, and metabolic parameters (glucose absorption [31,33,34], lactate excretion [32,33], urea [32,33], ATP level [35,36]), etc., are assessed. However, the experimental study of intracellular metabolism of an integral organ without disrupting its structure is difficult. Mathematical modeling is used to solve such problems. The use of modeling to obtain complex maps of metabolic fluxes in the liver is known [37]. Such quantitative maps have proven to be extremely useful for comparing the effects of various stress factors on organ metabolism [38,39,40], since they provide an overall picture and understanding of changes in the corresponding metabolic pathways [38]. However, the type of stress affecting an organ can vary significantly: it can be hypoxia, exposure to extreme temperatures, accumulation of toxic metabolites, etc. [41,42]. In this work, magnetic nanoparticles are considered as such a stress factor.

This study aims to evaluate the metabolic activity of the liver under ex vivo perfusion conditions exposed to magnetic nanoparticles using mathematical modeling. The plan is to obtain a picture of metabolic fluxes and assess how the presence of magnetic nanoparticles affects the distribution of energy resources in liver cells without impairing liver function.

## 2. Materials and Methods

### 2.1. Synthesis and Characterization of Magnetite Nanocomposites

Magnetite nanoparticles were obtained by coprecipitation at room temperature from a solution of Fe(NO_3_)_3_ (SRL, Mumbai, India) and FeSO_4_ (SRL, Mumbai, India) salts in a molar ratio of 2:1. The resulting samples were dissolved in distilled water and added to a solution of NH_4_OH (25%) (Chemreactivsnab, Izhevsk, Russia) with stirring until pH = 10 was reached. The stirring time was 30 min. After this time, the magnetite nanoparticles were collected using a magnet and washed in distilled water until pH = 7.0 was reached. 1 g of arabinogalactan (Ametis, Blagoveshchensk, Russia) was added to the obtained nanoparticles, and the suspension was treated with ultrasound for 10 min (22 kHz, 50 W/cm^2^). A detailed description of the synthesis method and the results of nanoparticle characterization are presented in reference [43].

### 2.2. Animals and Experimental Design

The experiments were performed on isolated perfused livers of male Wistar rats weighing 200–250 g (16 experimental animals). Perfusion was carried out using Krebs–Henseleit physiological medium under normothermic conditions (37 °C), and the perfusion medium was oxygenated with a gas mixture containing 95%O_2_/5%CO_2_.

Animals are divided into 2 groups:

- “Krebs–Henseleit buffer” (8 animals)–isolated liver perfused with Krebs–Henseleit buffer–control group;
- “Krebs–Henseleit buffer + Nanoparticles” (8 animals)–isolated liver perfused with Krebs–Henseleit buffer with the addition of magnetite nanoparticles (concentration in the buffer: 10 mg/mL)–experimental group.

Perfusion was performed using an isolated organ culture system with an “open” (non-recirculating) configuration, which implies a single-pass flow of the perfusion medium through the organ. The perfusion medium, either in its “pure” form for the control group or supplemented with magnetite nanoparticles for the experimental group, was first equilibrated with an appropriate gas mixture in an oxygenator. It was then delivered by a peristaltic pump to the organ, which was placed in a chamber with an air space isolated from the external environment. The perfusion medium, flowing through the “arterial” line, entered the liver via the portal vein, which functioned as the inflow vessel. After passing through the organ, the fluid exited via the inferior vena cava, which served as the “venous” outflow line.

The gas mixture was supplied to the chamber for the purpose of studying the delivery of oxygen through the surface using a pump from a container that was filled with a gas mixture, the main components of which were oxygen and carbon dioxide in a ratio of 95%/5%.

Liquid and gas samples were collected both before and after entering the organ chamber using a disposable syringe. A gas sample was also collected from the organ chamber using a 20 mL disposable syringe.

The organ isolation procedure was performed under general thiopental sodium anesthesia (100 mg/kg body weight). To prevent blood coagulation, heparin was administered intravenously into the femoral vein (10,000 IU/kg body weight). Subsequently, the abdominal cavity was opened, and cannulation of the portal vein (*v. portae*) and the common bile duct (*ductus choledochus*) was performed. The thoracic cavity was then opened, and cannulation of the inferior vena cava (*v. cava inferior*) was carried out, followed by liver excision from the rat [44].

The chamber in which the liver was placed was hermetically sealed and filled with a gas mixture, the rate of which remained constant throughout the perfusion–15 mL/min.

Perfusion was conducted under normothermic conditions (37 °C) at a constant perfusion flow rate of 2.5 mL/(min·g) (per gram of organ wet weight). The total perfusion time was 2 h. During the first 30 min, samples were collected every 10 min, and starting from the 30th minute, every 5 min. Organ viability was assessed based on vascular resistance (cm H_2_O), and perfusion medium samples were collected from both the “arterial” and “venous” lines to measure metabolite concentrations (glucose), electrolytes (K^+^, Na^+^), and blood gases (O_2_, CO_2_).

In addition, gas samples were collected every 15 min at the inlet to the organ chamber and from the chamber itself to determine the partial pressure of oxygen (mmHg). The partial pressure of oxygen in the perfusion fluid and in the chamber gas phase was measured using an amperometric method with a Clark electrode on an ABL800Flex analyzer (Radiometer, Brønshøj, Denmark). The volume of each gas sample was 20 mL. The volume of each perfusion fluid sample was 195 µL [45].

The above parameters, based on the differences between the “arterial” and “venous” lines, allowed the calculation of metabolic activity indicators, including:

- specific rates of oxygen uptake and carbon dioxide release through the vascular bed (µmol·min^−1^·g^−1^);
- specific rate of oxygen uptake through the liver surface (µmol·min^−1^·g^−1^);
- specific rates of sodium and potassium ion loss (µmol·min^−1^·g^−1^);
- specific rate of glucose uptake (µmol·min^−1^·g^−1^).

### 2.3. Determination of the Quantitative Content of Magnetite Nanoparticles in Samples

Perfusate sampling for determination of magnetite nanoparticle concentration in the “arterial” and “venous” lines was performed every 15 min during perfusion using inductively coupled plasma mass spectrometry (ICP-MS) (Agilent Technologies, Santa Clara, CA, USA).

### 2.4. Flux-Based Modeling of Hepatic Metabolism

Flux-based models constructed using experimental data obtained from isolated perfused livers are applied to analyze hepatic functional capacity. The hepatic metabolic network includes all major metabolic pathways of the liver, such as gluconeogenesis, glycolysis, the urea cycle, fatty acid metabolism, the pentose phosphate pathway, the Krebs cycle, and the metabolism of glycogen and amino acids. Orman et al. modified hepatic flux models to include glycolytic and gluconeogenic pathways, as well as pathways of fatty acid synthesis and oxidation, glycogenesis, and glycogenolysis [39]. This model is considered a small-scale model and includes 72 metabolites and 84 metabolic fluxes.

As the primary theoretical method of the study, flux modeling was used, specifically flux balance analysis (FBA) of steady-state metabolic fluxes.

### 2.5. Assessment of the Consistency of Energy Resource Distribution

The degree of consistency in the distribution of energy resources was assessed using the Zipf–Pareto distribution and its linear approximation, the Zipf–Pareto–Mandelbrot distribution [46].

The Zipf–Pareto distribution is an equation in double logarithmic coordinates that describes a regime of free competition for resources.

The consistency of energy resource distribution is determined by the coefficient of determination (R^2^) for each group. A value of the coefficient close to 1 indicates a more consistent distribution of energy resources and a higher degree of regulation.

If the R^2^ value was close to unity for the Zipf–Pareto distribution, the distribution of energy resources among metabolic fluxes was considered optimal. If the R^2^ value was closer to unity for the Zipf–Pareto–Mandelbrot distribution, the system was considered to be under the influence of an external factor.

### 2.6. Statistical Analysis

Statistical data processing was performed using Microsoft Excel (2021) spreadsheet software from the standard Microsoft Office suite and the R programming language.

The Shapiro–Wilk test was used to assess normality of distribution for small samples (*n* ≤ 50). Data were considered to follow a normal distribution at a significance level of *p* > 0.05. Normally distributed data are presented as the arithmetic mean ($\bar{x}$) and standard deviation (*σ*) in the form $\bar{x}$ ± *σ*. Non-normally distributed data are presented as the median (*Me*), first (*Q1*), and third (*Q3*) quartiles in the form *Me* [*Q1*; *Q3*].

Comparisons between experimental groups with normally distributed data were performed using the two-sample Student’s *t*-test for independent samples. For non-normally distributed data, the Mann–Whitney U test was applied. The significance level was set at 0.05 for both tests.

## 3. Results and Discussion

### 3.1. Dynamics of Liver Metabolic Activity Parameters

During the experiment, hepatic arterial pressure remained within normal values for both the control and experimental groups, ranging from 9 to 14 mm H_2_O throughout the entire perfusion period.

**Figure 1:**
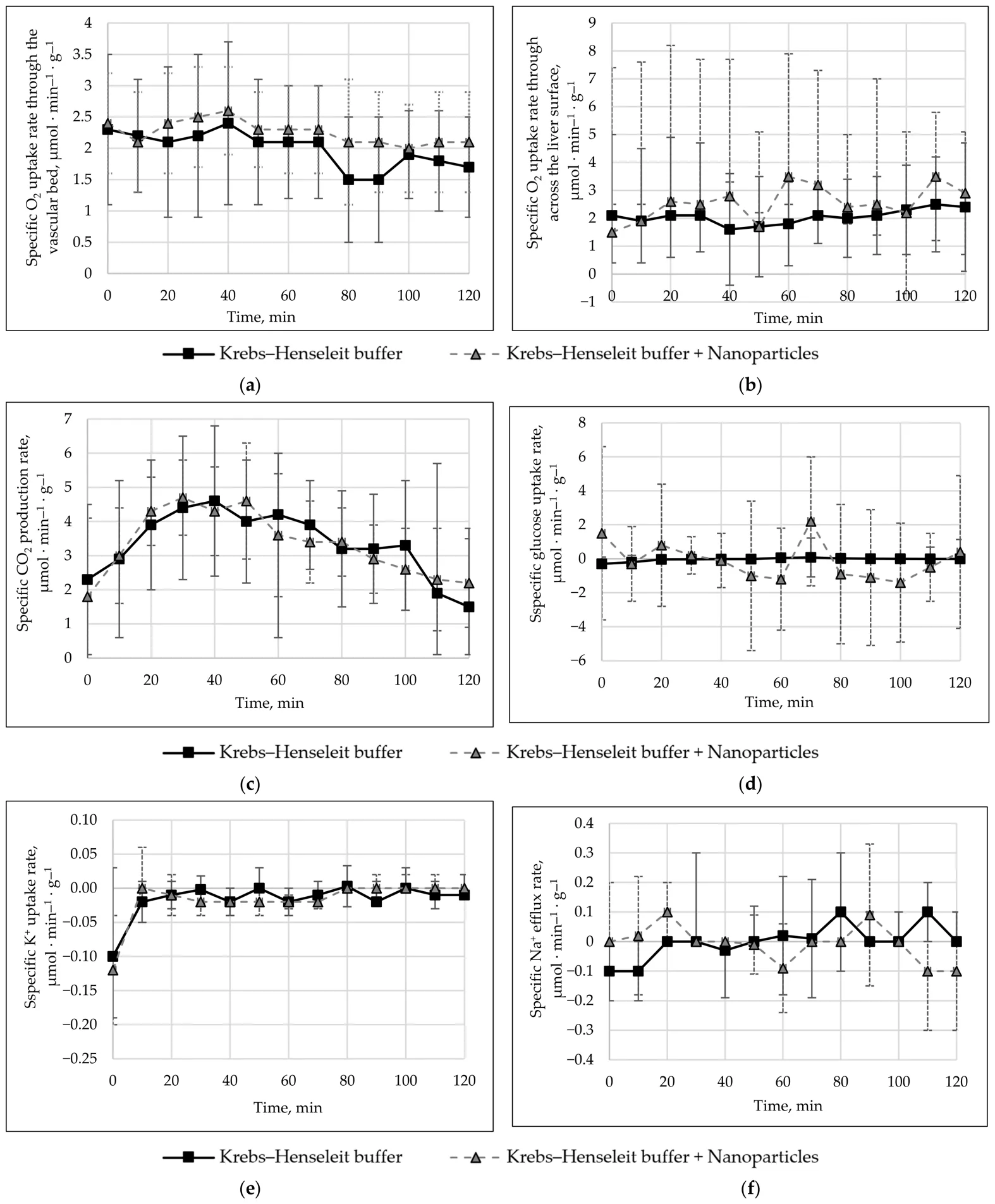
Parameters of liver metabolic activity. (**a**) Specific rate of oxygen uptake through the vascular bed. (**b**) Specific rate of oxygen uptake through the liver surface. (**c**) Specific rate of carbon dioxide release. (**d**) Specific rate of glucose uptake. (**e**) Specific rate of potassium uptake. (**f**) Specific rate of sodium release.

Figure 1 presents the metabolic activity parameters. The specific rates of oxygen uptake through the vascular bed in both experimental groups remained nearly unchanged throughout perfusion, averaging 1.99 µmol·min^−1^·g^−1^ in the control group and 2.25 µmol·min^−1^·g^−1^ in the nanoparticle-treated group (Figure 1a).

During the perfusion period, the rate of oxygen uptake through the liver surface in the experimental group was higher than in the control group, averaging 2.55 µmol·min^−1^·g^−1^ and reaching up to 3.5 µmol·min^−1^·g^−1^ during the 60–70 min interval of perfusion, whereas in the control group the uptake rate remained stable, averaging 2.00 µmol·min^−1^·g^−1^ (Figure 1b).

For the dynamic curves of the specific carbon dioxide production rate, both groups showed a gradual increase in this parameter up to the 50th minute, from 2.0 µmol·min^−1^·g^−1^ to 4.3 µmol·min^−1^·g^−1^ (Figure 1c). By the end of perfusion, these rates returned almost to their initial values.

The specific rate of glucose uptake in the control group remained stable and showed virtually no changes (Figure 1d). In the nanoparticle-treated group, the rate remained relatively stable up to the 40th minute, after which pronounced fluctuations were observed, including sharp increases (at 60 and 100 min) and decreases (at 40 and 70 min).

The specific rates of potassium uptake in both groups, starting from the 10th minute of perfusion, continuously fluctuated around a value of approximately −0.01 µmol·min^−1^·g^−1^ (Figure 1e), while remaining relatively stable throughout the perfusion period.

The specific rate of sodium release in the control group gradually increased from 0 to 20 min, then remained stable until the 70th minute, after which sharp increases were observed, reaching up to 1 µmol·min^−1^·g^−1^ at the 80th and 100th minutes of perfusion (Figure 1f). In the experimental group with nanoparticles in the perfusate, gradual fluctuations were observed, with values ranging between 1.0 µmol·min^−1^·g^−1^ and −1.0 µmol·min^−1^·g^−1^.

Despite some deviations in the values between the control and experimental groups, no statistically significant differences were observed for any of the parameters. These values are consistent with literature data and indicate normal liver function [47,48,49].

This indicates that the presence of magnetite nanoparticles in the perfusion medium does not affect the metabolic activity parameters of the liver. Therefore, it is necessary to confirm the uptake of magnetite nanoparticles by the liver during perfusion.

### 3.2. Quantitative Content of Magnetite Nanoparticles in the Liver

Table 1 presents the concentration values of magnetite nanoparticles during perfusion in the inflow and outflow, as well as the specific rate of magnetite nanoparticle uptake by the liver. The concentration of nanoparticles at the liver inlet remained relatively stable throughout the experiment, varying within the range of 3.05–3.48 mg/mL, indicating a constant supply of nanoparticles to the system. During the first 45 min of perfusion, uptake remained at a relatively low and stable level, accounting for 20–26%. Starting from the 60th minute, a sharp increase in uptake was observed, reaching 37%, after which the parameter continued to rise progressively, reaching 52% by the 90th minute and plateauing at 54% at 105–120 min. Thus, the maximum uptake of nanoparticles by the liver under isolated perfusion conditions was 54% of the administered dose, while a significant fraction of nanoparticles remained in the perfusate, indicating saturation of cellular clearance mechanisms.

The uptake of nanoparticles from the perfusate by isolated liver is a multifactorial process based on several cellular mechanisms. The main effectors of uptake are liver macrophages, Kupffer cells, which are located in the lumen of the sinusoids. It has been shown that these cells are the main barrier determining the fate of nanoparticles in the liver, and their phagocytic activity leads to rapid saturation of the uptake system [50]. In parallel, the elimination process involves endothelial cells of the sinusoids, which express scavenger receptors that ensure receptor-mediated clearance of particles from the bloodstream [51]. The contribution of the formation of a protein corona on the surface of nanoparticles cannot be excluded. It is reported that such a corona is capable of imparting a new biological identity to the particle, facilitating its recognition by cells; however, this mechanism was not separately studied in our work and is considered only as a probable factor [52].

The measured nanoparticle uptake concentration confirms their presence in the liver. This also did not affect changes in intracellular metabolite concentrations. This suggests that simply measuring intracellular parameters is insufficient to study the effect of nanoparticles on organ metabolism. Therefore, mathematical modeling methods should be used to analyze intracellular processes based on extracellular parameters.

### 3.3. Mathematical Modeling of the Liver Under Ex Vivo Conditions

To identify metabolic features of the isolated perfused liver with and without magnetic nanoparticles, metabolic flux distributions were used.

The liver metabolic model includes glycolysis, gluconeogenesis, the respiratory chain, the pentose phosphate pathway, amino acid metabolism, lipid metabolism, the Krebs cycle, glycogen metabolism, and the ornithine cycle. In total, 72 metabolites and 84 metabolic fluxes were considered [39], including the oxygen uptake flux through the liver surface [47,48,49]:

2ATP → 2ADP + 2Pi + O_2_,

ATP—adenosine triphosphate; ADP—adenosine diphosphate; Pi—inorganic phosphate;
O_2_—oxygen.

Based on reaction, substrate, and product data, a metabolic network is constructed and represented as a stoichiometric matrix, where each row corresponds to a metabolite and each column corresponds to a reaction (Table S1).

However, the number of reactions in metabolic pathways exceeds the number of metabolites, which makes the system of equations underdetermined. To address this problem, linear programming can be applied with constraints on metabolic fluxes and with objective functions defined as maximization of one or several fluxes. The calculated first and third quartiles can be used as bounds for these constraints (Table 2).

**Figure 2:**
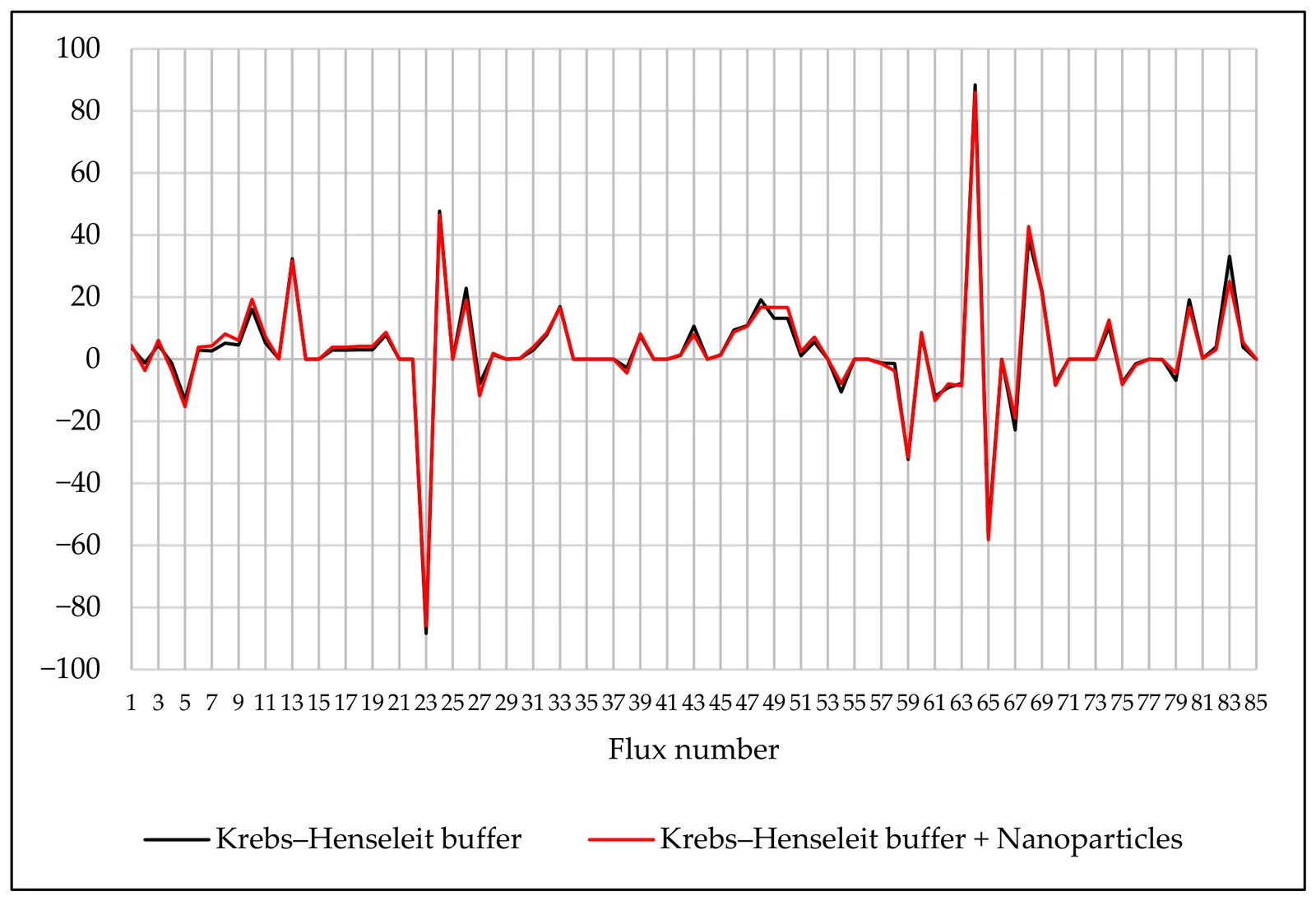
Values of metabolic fluxes in the isolated perfused rat liver under normal conditions and under exposure to magnetic nanoparticles. 1–13—glycolysis, gluconeogenesis, pentose phosphate pathway; 14–19—Krebs cycle; 20–22—ornithine cycle; 23–42—amino acid metabolism; 43–48—lipid metabolism; 49–50—glycogen metabolism; 51–52—respiratory chain; 53–83—exchange reactions; 84–85—oxygen exchange through the liver surface.

As the objective function, ATP production by liver cells was chosen to be maximized [53]. Figure 2 presents the metabolic flux distribution graph for both groups.

The introduction of magnetic nanoparticles leads to changes in hepatic metabolic fluxes as well as a redistribution of cellular energy resources. These changes affect processes such as glycolysis, gluconeogenesis, the respiratory chain, and oxygen exchange through the liver surface.

The first 12 reactions associated with glycolysis, gluconeogenesis, and the pentose phosphate pathway proceed more actively in the experimental group with magnetite nanoparticles. Fluxes 2 (production of glucose-6-phosphate), 5 (production of glyceraldehyde-3-phosphate), 6 (formation of phosphoenolpyruvate), 8 (formation of glucose-6-phosphate), and 9 (production of fructose-1,6-bisphosphate) are below 0, indicating that the reactions proceed in the direction of glycolysis.

Flux 13, responsible for the conversion of lactate to pyruvate, remains unchanged and positive in both groups.

Krebs cycle reactions (fluxes 14–19) are active in both groups; however, in the experimental group they proceed slightly faster, which is also reflected in fluxes 51 and 52 responsible for the electron transport process.

Urea cycle fluxes (20–22) are active only in flux 20, which describes the conversion of arginine into urea and ornithine; this reaction also proceeds more actively in the experimental group.

Amino acid metabolism (fluxes 23–42) shows almost no differences between the groups, except for the reactions of serine and aspartate formation in fluxes 27 and 38, which proceed more actively in the experimental group.

Lipid metabolism (fluxes 43–48) also differs slightly between the groups. Flux 43 is higher in the control group, indicating lower palmitate uptake under the influence of nanocomposites. A similar pattern is observed for flux 48 in the experimental group, where the flux value is lower, indicating reduced uptake of β-hydroxybutyric acid.

Glycogen metabolism (fluxes 49–50) proceeds more actively in both directions in the experimental group; however, a futile cycle is formed due to increased activity of glycogen synthesis and degradation reactions.

The analysis of intracellular liver metabolism under ex vivo conditions showed that metabolic fluxes in the two groups differ only slightly, leading to a redistribution of cellular energy resources without causing a complete disruption of metabolic processes. However, the introduction of nanoparticles increases the activity of certain reactions, such as glycolysis, the Krebs cycle, glycogen metabolism, and the respiratory chain.

These changes in reactions associated with ATP consumption require further investigation regarding the consistency of energy resource distribution within liver cells. For this purpose, Pareto methodology can be used to assess optimal resource allocation.

### 3.4. Assessment of the Efficiency of Intracellular Metabolic Processes in the Liver Under Ex Vivo Conditions

To assess the degree of consistency in resource distribution, the Zipf–Pareto distribution and its linearized form, the Zipf–Pareto–Mandelbrot distribution, were applied.

For constructing a model of energy resource distribution in the isolated perfused liver, the obtained values of metabolic fluxes are used.

The model of energy resource distribution is constructed separately for each group. From all metabolic flux values, only those reactions are selected that require the presence of macroergic compounds–ATP and ADP–for their progression. Thus, the following fluxes were included in the construction of the energy resource distribution model: the glucokinase reaction, the reaction chain converting glucose-6-phosphate to pyruvate, the reaction chain converting pyruvate to glucose-6-phosphate, glycogen synthesis, the Krebs cycle, the ornithine cycle, the electron transport chain, ATP hydrolysis, and oxygen uptake through the liver surface.

**Figure 3:**
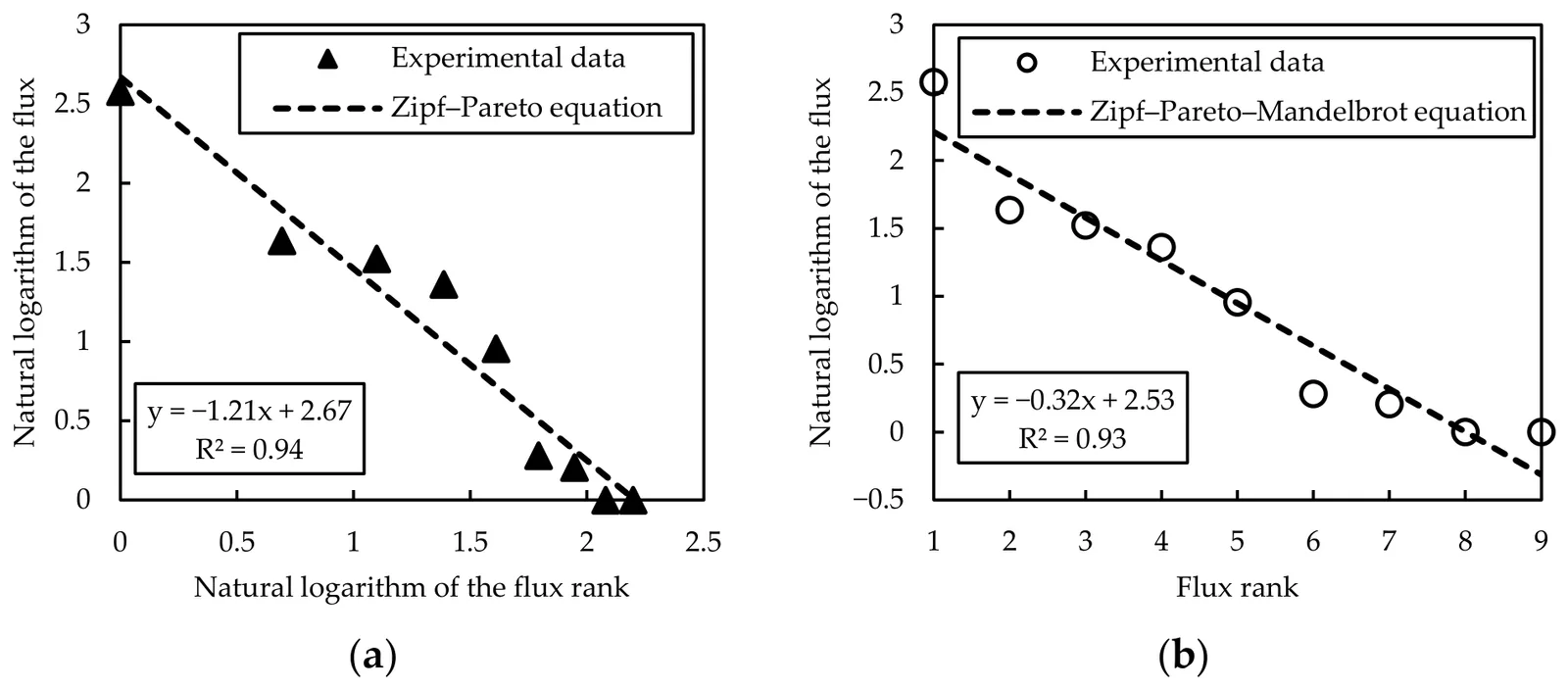
Distribution of metabolic fluxes for the “Krebs–Henseleit buffer” group. (**a**) Zipf–Pareto equation. (**b**) Zipf–Pareto–Mandelbrot equation.

The distribution of metabolic fluxes for the “Krebs–Henseleit buffer” group using the Zipf–Pareto equation and the Zipf–Pareto–Mandelbrot approximation is presented in Figure 3. The lower left corner of each graph also shows the approximation equations, where y represents the natural logarithm of the flux and x represents the natural logarithm of the flux rank (for the Zipf–Pareto equation) or the flux rank (for the Zipf–Pareto–Mandelbrot equation).

It can be seen from Figure 3 that the experimental data are better described by the Zipf–Pareto equation, since the Spearman rank correlation coefficient for the Zipf–Pareto model is 0.94, which is higher than that of the Zipf–Pareto–Mandelbrot model.

**Figure 4:**
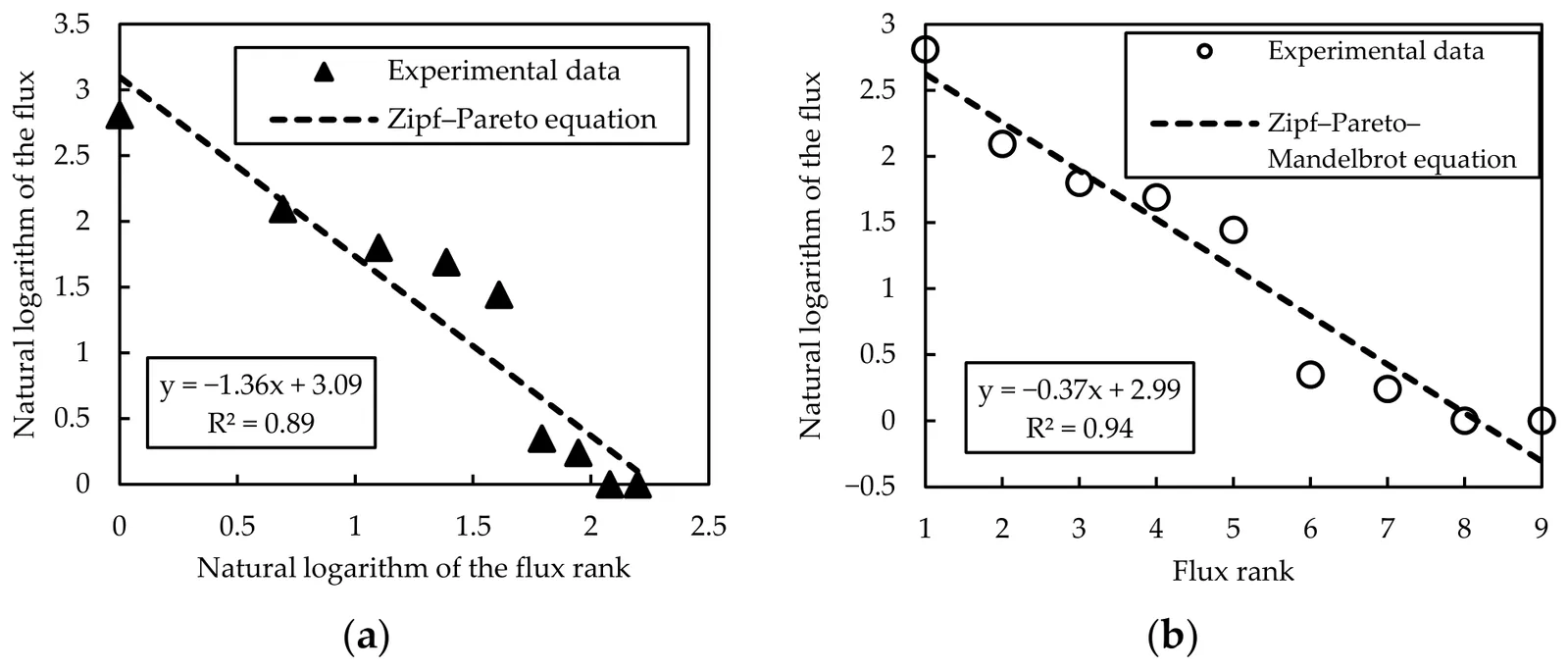
Distribution of metabolic fluxes for the “Krebs–Henseleit buffer + nanoparticles” group. (**a**) Zipf–Pareto equation. (**b**) Zipf–Pareto–Mandelbrot equation.

The distribution of metabolic fluxes for the “Krebs–Henseleit buffer + nanoparticles” group using the Zipf–Pareto equation and the Zipf–Pareto–Mandelbrot equation is presented in Figure 4.

From Figure 4, the experimental data are better described by the Zipf–Pareto–Mandelbrot equation, since the Spearman rank correlation coefficient is higher than that of the Zipf–Pareto model (R^2^ = 0.94 vs. R^2^ = 0.89).

The Zipf–Pareto and Zipf–Pareto–Mandelbrot equations provide a good approximation of the rank distribution data.

The first equation was used to describe the state of the liver under normal conditions; thus, competition between metabolic fluxes (consumers) occurs for a single resource.

The second equation was applied to describe the state of the liver under exposure to nanoparticles. In this case, competition between metabolic fluxes occurs either for several interrelated resources or under the influence of external factors acting on the system.

High values of the coefficient of determination for the Zipf–Pareto equation in the “Krebs–Henseleit buffer” group indicate that, under normal conditions, the metabolic system of the isolated organ operates optimally, without dysregulation; the energy resource is distributed most efficiently, and competition occurs for a single energy resource.

When studying the “Krebs–Henseleit buffer + Nanoparticles” group, the value of the determination coefficient also turns out to be high, but the rank distribution of metabolic flows is described by the Zipf–Pareto–Mandelbrot equation, which can be explained by the presence of an external extreme effect–the effect of nanoparticles, which does not introduce a mismatch into the system.

## 4. Conclusions

The study showed that the introduction of magnetic nanocomposites changes metabolic flows (glycolysis, respiratory chain, oxygen exchange through the surface) using the energy of ATP and ADP and therefore leads to a redistribution of energy resources in cells, which undoubtedly requires an assessment of the consistency of this distribution.

This assessment was conducted using the Zipf–Pareto method and its linear approximation, the Zipf–Pareto–Mandelbrot method. Here, the energy stored in hepatocytes in the form of high-energy compounds was considered a resource, and metabolic flows were considered consumers of this resource. The results demonstrated the phenomenon of “free competition” for the resource, i.e., energy was optimally distributed among all metabolic flows, both under normal conditions and in the presence of nanoparticles.

The obtained data indicate that magnetic nanoparticles have the potential to be used in transplantology and cryopreservation, as they affect liver metabolism without significantly impairing its functional state. Importantly, almost half of the nanoparticles remained in the perfusate, indicating the ability of the developed particles to partially bypass hepatic clearance and not be completely taken up by organ tissue. This may be particularly useful in ex vivo perfusion systems, where it is necessary to maintain a controlled concentration of the functional agent in the circulating medium throughout the procedure. In cryopreservation settings, such particles can be considered a promising component of technologies aimed at more uniform cooling and subsequent controlled heating of the organ. Thus, magnetic nanocomposites are of interest for further investigation as a possible element of strategies for preparing donor livers for transplantation and long-term storage. Overall, an integrated approach combining experimental perfusion, analytical measurement, and mathematical modeling of metabolic fluxes appears to be very promising for studying the action of nanoparticles and predicting their impact on liver function.

## Figures and Tables

**Table 1:** Concentration of magnetite nanoparticles in the perfusate and liver uptake over time.

Time, min	Inflow (mg/mL)	Outflow (mg/mL)	ΔC (mg/mL)	Uptake, % *
0	3.15 ± 0.15	2.32 ± 0.03	0.83	26%
15	3.43 ± 0.10	2.64 ± 0.04	0.78	23%
30	3.48 ± 0.10	2.79 ± 0.09	0.69	20%
45	3.24 ± 0.17	2.56 ± 0.14	0.68	21%
60	3.40 ± 0.09	2.13 ± 0.12	1.26	37%
75	3.19 ± 0.08	1.83 ± 0.10	1.35	43%
90	3.41 ± 0.06	1.65 ± 0.17	1.76	52%
105	3.21 ± 0.11	1.46 ± 0.16	1.74	54%
120	3.05 ± 0.10	1.39 ± 0.11	1.65	54%

* Uptake was calculated as (Inflow − Outflow)/Inflow × 100%.

**Table 2:** Concentration of magnetite nanoparticles in the perfusate and hepatic uptake.

Flux	Flux Number	Krebs–Henseleit Buffer		Krebs–Henseleit Buffer + Nanoparticles	
		Lower Bound	Upper Bound	Lower Bound	Upper Bound
Oxygen (surface)	84	0.49	4.46	0.99	6.09
Oxygen (vascular bed)	81	0.11	0.29	0.11	0.32
Carbon dioxide	82	1.27	6.37	0.65	5.48

## Data Availability

The data presented in this study is available on request from the corresponding author.

## Supplementary Materials

The following supporting information can be downloaded at: https://www.mdpi.com/article/10.3390/biomedicines14091877/s1, Table S1: Hepatic Network Model.
