# Supplementary material for: Mathematical Modeling of Liver Metabolic Activity Under Ex Vivo Conditions upon Exposure to Magnetic Nanoparticles
Source: Biomedicines. 2026 Aug 22;14(9):1877. doi: 10.3390/biomedicines14091877 (PMC13604296; doi:10.3390/biomedicines14091877)
Supplement: Supplementary file 1 [file biomedicines-14-01877-s001.zip › biomedicines-4428371-supplementary.pdf]

Table S1. Hepatic Network Model.

Reaction no	Enzymes and explanations	Glycolysis & Gluconeogenesis
Reaction 1	Glucose-6-Pase	Glucose-6-P + H2O ==> Glucose + Pi
Reaction 2	Phosphoglucose isomerase	Fructose-6-P <==> Glucose-6-P
Reaction 3	Fructose-1,6-Pase-1	Fructose-1,6-P2 + H2O ==> Fructose-6-P + Pi
Reaction 4	Triose P-isomerase, fructose biphosphate aldolase	2 Glyceraldehyde-3-P <==> Fructose-1,6-P2
Reaction 5	Glyceraldehyde-P dehydrogenase, 3-phosphoglycerate kinase, phosphoglyceromutase, enolase	ATP + NADH + PEP + H+ + H2O <==> Glyceraldehyde-3-P + Pi + NAD+ + ADP
Reaction 6	PEPCK	Oxaloacetate + GTP <==> CO2 + PEP + GDP
Reaction 7	Pyruvate carboxylase	CO2 + ATP + Pyruvate + H2O ==> Pi + ADP + Oxaloacetate
Reaction 8	Hexokinase	Glucose + ATP ==> Glucose-6-P + ADP
Reaction 9	PFK-1	Fructose-6-P + ATP ==> Fructose-1,6-P2 + ADP
Reaction 10	Pyruvate kinase	ADP + PEP ==> ATP + Pyruvate
Reaction 11	PDH	NAD+ + Pyruvate + CoA-SH ==> CO2 + NADH + Acetyl-CoA + H+
		PPP
Reaction 12	Glucose-6-P dehydrogenase and 3 additional steps	Glucose-6-P + 12 NADP+ + 7 H2O ==> 6 CO2 + 12 NADPH + Pi + 12 H+
		Lactate
Reaction 13	Lactate dehydrogenase	NAD+ + Lactate <==> NADH + Pyruvate + H+
		TCA
Reaction 14	Citrate synthase	Oxaloacetate + Acetyl-CoA + H2O ==> Citrate + CoA-SH + H+
Reaction 15	Aconitase, isocitrate dehydrogenase	NAD+ + Citrate <==> CO2 + NADH + a-ketoglutarate

Reaction 16	a-ketoglutarate dehydrogenase	NAD+ + CoA-SH + a-ketoglutarate ==> CO2 + NADH + Succinyl-CoA + H+
Reaction 17	Succinyl-CoA synthase and succinate dehydrogenase	Pi + GDP + Succinyl-CoA + FAD <==> GTP + CoA-SH + Fumarate + FADH2
Reaction 18	Fumarase	Fumarate + H2O <==> Malate
Reaction 19	Malate dehydrogenase	NAD+ + Malate <==> NADH + Oxaloacetate + H+
		Urea
Reaction 20	Arginase	Arginine + H2O ==> Urea + Ornithine
Reaction 21	Carbonate dehydratase, carbamoyl-P synthase, ornithine transcarbamylase	CO2 + 2 ATP + Ornithine + NH4+ + H2O <==> 2 Pi + 2 ADP + Citrulline + 3 H+
Reaction 22	Argininosuccinate synthetase, argininosuccinase.	ATP + Citrulline + Aspartate ==> Fumarate + Arginine + AMP + PPi
		Amino acid metabolism
Reaction 23	Alanine aminotranferase	NAD+ + Alanine + H2O <==> NADH + Pyruvate + NH4+ + H+
Reaction 24	Serine dehydratase	Serine ==> Pyruvate + NH4+
Reaction 25	Transaminase, 3-mercaptopyruvate sulfurtransferase	NAD+ + Cysteine + H2SO3 + H2O <==> NADH + Pyruvate + NH4+ + H2S2O3 + H+
Reaction 26	Threonine 3-dehydrogenase, acetyl-CoA ligase	NAD+ + CoA-SH + Threonine ==> NADH + Acetyl-CoA + Glycine
Reaction 27	Glycine hydroxymethyltranferase, glycine cleavage system	NAD+ + 2 Glycine <==> CO2 + NADH + NH4+ + Serine
Reaction 28	Lysine metabolism (8 steps)	5 NAD+ + CoA-SH + FAD + Lysine + 3 H2O ==> 2 CO2 + 5 NADH + FADH2 + 2 NH4+ + Acetoacetyl-CoA + 5 H+
Reaction 29	Phenylalanine hydroxylase	Phenylalanine + Tetrahydrobiopterin + O2 ==> Dihydrobiopterin + Tyrosine + H2O
Reaction 30	Tyrosine metabolism (5 steps)	NAD+ + 2 O2 + Tyrosine + H2O ==> CO2 + NADH + Fumarate + NH4+ + H+ + Acetoacetate

Reaction 31	Glutamate dehydrogenase	NAD+ + Glutamate + H2O <==> NADH + a-ketoglutarate + NH4+ + H+
Reaction 32	Glutaminase	Glutamine + H2O ==> NH4+ + Glutamate
Reaction 33	Ornithine metabolism (2 steps)	NADP+ + NAD+ + Ornithine + H2O ==> NADPH + NADH + NH4+ + Glutamate + H+
Reaction 34	Proline oxidase, 1-pyrroline-5-carboxylate dehydrogenase	0.5 NADP+ + 0.5 NAD+ + 0.5 O2 + Proline ==> 0.5 NADPH + 0.5 NADH + Glutamate + H+
Reaction 35	Histidine metabolism (4 steps)	Histidine + THF + 2 H2O ==> NH4+ + Glutamate + 2-formimino-THF
Reaction 36	Methionine metabolism (5 steps)	ATP + NAD+ + CoA-SH + Serine + Methionine ==> CO2 + Pi + NADH + NH4+ + Cysteine + PPi + Adenosine + Propinoyl-CoA
Reaction 37	Propinoyl-CoA carboxylase, Methylmalonyl-CoA epimerase, Methylmalonyl-CoA mutase	CO2 + ATP + Propinoyl-CoA ==> Succinyl-CoA + AMP + PPi
Reaction 38	Aspartate aminotransferase	NAD+ + Aspartate + H2O <==> NADH + Oxaloacetate + NH4+ + H+
Reaction 39	Asparaginase	Asparagine + H2O ==> NH4+ + Aspartate
Reaction 40	Valine metabolism (7 steps)	0.5 NADP+ + 3.5 NAD+ + FAD + 2 H2O + valine ==> 2 CO2 + 0.5 NADPH + 3.5 NADH + FADH2 + NH4+ + Propinoyl-CoA + 3 H+
Reaction 41	Isoleucine Metabolism (6 steps)	0.5 NADP+ + 2.5 NAD+ + FAD + 2 H2O + isoleucine ==> CO2 + 0.5 NADPH + 2.5 NADH + Acetyl-CoA + FADH2 + NH4+ + Propinoyl-CoA + 3 H+
Reaction 42	Leucine Metabolism (6 steps)	0.5 NADP+ + ATP + 1.5 NAD+ + FAD + H2O + leucine ==> 0.5 NADPH + Pi + ADP + 1.5 NADH + Acetyl-CoA + FADH2 + NH4+ + 2 H+ + Acetoacetate
		Lipid

Reaction 43	Hepatic Lipase, Glycerol-3-P dehydrogenase	Palmitoylglycerol + NAD+ + 3 H2O <==> Glyceraldehyde-3-P + 3 Palmitate + NADH + 4 H+
Reaction 44	Fatty acid oxidation (7x4 steps)	ATP + 7 NAD+ + Palmitate + 8 CoA-SH + 7 FAD ==> 2 Pi + 7 NADH + 8 Acetyl-CoA + 7 FADH2 + AMP
Reaction 45	Fatty acid synthesis (7x4 steps)	14 NADPH + 7 ATP + 8 Acetyl-CoA + 14 H+ ==> 14 NADP+ + 7 Pi + Palmitate + 7 ADP + 6 H2O
Reaction 46	Thiolase (Ketogenesis)	2 Acetyl-CoA <==> 2 CoA-SH + Acetoacetyl-CoA
Reaction 47	HMG-CoA synthase and lyase (Ketogenesis)	Acetoacetyl-CoA + H2O ==> CoA-SH + Acetoacetate
Reaction 48	B-OH-butyrate dehydrogenase (Ketogenesis)	NADH + H+ + Acetoacetate <==> NAD+ + B-OH-butyrate
		Glycogen
Reaction 49	Glycogenolysis	Glucose-6-P + ATP + H2O + Glycogen(n-1) ==> ADP + Glycogen
Reaction 50	Glycogenesis	Pi + Glycogen ==> Glucose-6-P + Glycogen(n-1)
		Electron
Reaction 51	Electron transport system	3 ADP + NADH + 0.5 O2 + H+ ==> 3 ATP + NAD+ + H2O
Reaction 52	Electron transport system	2 ADP + FADH2 + 0.5 O2 ==> 2 ATP + FAD + H2O
		Exchange
Reaction 53		Glycogen <==>
Reaction 54		Palmitoylglycerol <==>
Reaction 55		valine <==>
Reaction 56		isoleucine <==>
Reaction 57		leucine <==>
Reaction 58		Glucose <==>
Reaction 59		Lactate <==>

Reaction 60		Urea <==>
Reaction 61		NH4+ <==>
Reaction 62		Ornithine <==>
Reaction 63		Arginine <==>
Reaction 64		Alanine <==>
Reaction 65		Serine <==>
Reaction 66		Cysteine <==>
Reaction 67		Threonine <==>
Reaction 68		Glycine <==>
Reaction 69		Glutamate <==>
Reaction 70		Glutamine <==>
Reaction 71		Proline <==>
Reaction 72		Histidine <==>
Reaction 73		Methionine <==>
Reaction 74		Aspartate <==>
Reaction 75		Asparagine <==>
Reaction 76		Lysine <==>
Reaction 77		Phenylalanine <==>
Reaction 78		Tyrosine <==>
Reaction 79		Acetoacetate <==>
Reaction 80		B-OH-butyrate <==>
Reaction 81		O2 <==>
Reaction 82		CO2 <==>
